# Effects of α-Ketoglutarate Peripartum Supplementation on Reproductive, Lactational, Productive and Immunological Outcomes in Dairy Cows

**DOI:** 10.3390/ani15081110

**Published:** 2025-04-11

**Authors:** Peng Wang, Xin Hu, Xiang’ao Shan, Jiarui Gao, Fei Guo, Bingyuan Wang, Guoshi Liu

**Affiliations:** 1 Beijing Key Laboratory for Animal Genetic Improvement, Key Laboratory of Animal Genetics and Breeding of the Ministry of Agricultural, State Key Laboratory of Animal Biotech Breeding, National Engineering Laboratory for Animal Breeding, College of Animal Science and Technology, China Agricultural University, Beijing 100193, China; 2 Beijing Changping District Animal Disease Prevention and Control Centre, Beijing 102200, China

**Keywords:** periparturient dairy cows, α-ketoglutarate, reproductive performance, lactation performance, immunity and antioxidation

## Abstract

In dairy cow, the peripartum metabolic stage is critical as may affect post-partum metabolic and reproductive recovery, colostrum quality, and overall reproductive fitness. This study aimed to evaluate the effects of varying doses of α-ketoglutarate (AKG) on lactation performance, reproductive performance, immune function, and antioxidant capacity in periparturient dairy cows. A total of 180 periparturient dairy cows were randomly assigned to four groups, with each cow receiving 1 g, 5 g, or 10 g of AKG in their prepartum diets. Results indicated that feeding 5 g and 10 g of AKG significantly increased the colostrum fat and protein content, reduced somatic cell counts, and improved daily milk yield. Regarding reproduction, AKG supplementation regulated reproductive hormones, increased postpartum estrogen levels, improved conception rates, and shortened the interval between breeding periods. For immune and antioxidant functions, AKG significantly increased serum IL-10 levels while reducing TNF-α and interleukins 1β and 6. It also significantly elevated glutathione peroxidase (GSH-PX) levels, reducing oxidative stress and demonstrating anti-inflammatory and immunomodulatory effects. Additionally, cows receiving medium-to-high doses of AKG had a significantly lower incidence of postpartum diseases such as mastitis. In conclusion, appropriate AKG supplementation can improve lactation performance, reproductive performance, immune function, and overall health in periparturient dairy cows, providing a theoretical basis for its use in dairy cow nutrition management.

## Simple Summary

In the current study, we investigated the potential effects of α-ketoglutarate (AKG) supplementation during the periparturient period on the reproductive physiology of dairy cows. The study included a total of 180 periparturient dairy cows and different doses of AKG supplementation. The results showed that AKG supplementation significantly increased the calf birth weight and shortened the interval between the postpartum period to next the successful breeding period, and that it lowered the incidence of postpartum diseases such as mastitis compared to the control group. It significantly improved the milk quality, including increases in the milk fat and protein contents and a decrease in the milk somatic cell count, but it also increased the daily milk yield. Mechanistic studies suggested that the beneficial effects of AKG supplementation during the periparturient period on reproductive health were attributed to AKG improving the immune function, antioxidant capacity, reproductive hormone secretion, and energy metabolism of mother cows.

## 1. Introduction

The periparturient period usually includes the three weeks of pre- and post-parturition, and during this period, cows will experience the most dramatic physiological adaptations [1]. For example, hepatic gluconeogenesis in maternal cows is accelerated, with a significant increase in fat mobilization and protein metabolism, accompanied by a dramatically increased metabolic burden which leads to a significant rise in energy demand [2,3]. Meanwhile, the dry matter intake in mother cows is reduced by more than 30% during the first 3 weeks of calving and this reduces nutrient and energy availability, putting the cow in a state of Negative Energy Balance (NEB) [4]. The NEB exacerbates their fat mobilization, leading to elevated levels of non-esterified fatty acids (NEFAs) and ketone bodies, which increases the risk of postpartum diseases and affects their lactation, reproductive performance, and overall health [5,6,7]. In addition, periparturient metabolic disorders may trigger an enhanced inflammatory response and impaired immune function, which in turn may increase the incidence of postpartum diseases such as mastitis and uteritis [8,9,10]. In order to cope with the increased energy demand and to enhance maternal immune function, cows require specific nutritional diets to enhance their energy intake and to ensure optimal pre- and postpartum performance.

Alpha-ketoglutarate (AKG) is a key intermediate in the tricarboxylic acid cycle, playing a central role in energy metabolism and the maintenance of normal metabolic processes in mammals [11]. Studies have shown that AKG is not only involved in energy metabolism, but also has multiple roles in enhancing immune function, increasing the amount of antioxidants, and promoting protein synthesis [12,13,14]. In animal studies, AKG supplementation to fresh farrowing sows increased the lactose and calcium content of their breast milk, which significantly enhanced the growth performance of their offspring, and also reduced lipopolysaccharide-induced intestinal stress injury in early weaned piglets and reduced the indices of piglet diarrhea [15,16]. In addition, in lambs with an AKG-enriched diet, AKG significantly increased the content of nitrogen in their feces and urine, reduced the proportion of ammonia in their sera, and also increased their bone density and strength as well as their survival rate compared to the control group [17,18]. There is a lack of studies in which AKG has been directly targeted at dairy cows, but studies of bovine in vitro embryo production have shown that appropriate concentrations of AKG improve egg mound dilation, oocyte quality, and embryo development [19]. To fill this research gap, this study was the first to investigate the effects of AKG in periparturient dairy cows.

Given the unique physiological demands of periparturient cows and their metabolic challenges, this study was designed to evaluate the potential effects of periparturient AKG supplementation on the physiological adaptations of cows, including their lactation performance, blood biochemical indices, and their general health. Regarding the supplementation of AKG to the diets of dairy cows, we expect this treatment will effectively alleviate the metabolic stress of cows caused by the NEB during the periparturient period and will therefore improve the lactation efficiency and reduce the risk of postpartum diseases and increase the overall health of cows. The results will provide valuable novel information regarding the use of AKG supplementation for the nutritional management of periparturient dairy cows.

## 2. Materials and Methods

Alpha-ketoglutarate (AKG) (purchased from Wuhan Camike Biomedical Technology Co., Ltd., Wuhan, China) was used to feed Holstein cows. Disposable syringes (10 mL) (Jiangsu Zhiyu Medical Equipment Co., Taizhou, China) were employed to collect tail vein blood samples from Holstein cows. Blood serum separation gel tubes (10 mL) (Hebei Kangwei Shi Medical Technology Co., Shijiazhuang, China) were utilized for the collection and separation of cow sera. Centrifuge tubes (50 mL) (Wuxi Nexus Life Science & Technology Co., Wuxi, China) were used to store milk samples, with potassium dichromate (Beijing Chemical Factory, Beijing, China) added to these tubes to prevent milk spoilage. Additionally, 2 mL centrifuge tubes (Changde Beekman Biotechnology Co., Changde, China) were used to store cow serum samples.

### 2.1. Experimental Design and Animal Management

This study was conducted on a commercial farm (Nankou San Farm) located in Beijing, China. One hundred and eighty healthy multiparous dairy periparturient Holstein cows with a body condition score (BCS) between 3 and 4 were selected for the study. Cows were clinically examined prior to the start of the trial to confirm their good health status. These cows were randomly divided into four groups of 45 for each group. The control group was fed a basal diet, while the experimental group was supplemented with 1 g, 5 g, and 10 g of AKG per cow per day, according to the expected date of parturition estimated by the farm computer system. AKG was dosed and mixed in a TMR mixer starting from 21 days before the planned parturition date and was administered until the day of delivery, after which it was stopped. Time to delivery did not differ significantly between groups. The study was approved by China Agricultural University and the protocol number was AW13014202-1-10.

### 2.2. Sample Collection and Processing

Blood samples were collected from 48 cows, with 12 randomly selected from each group. Samples were taken from the tail root vein on days 14 and 7 before parturition, within 2 h after calving, and on days 4, 7, and 14 postpartum. The blood samples were centrifuged at 1000× *g* for 10 min, and the supernatant was collected and stored at −20 °C for future analysis. Total protein, albumin, alanine aminotransferase, glutamine aminotransferase, total cholesterol, triglyceride, urea nitrogen, glucose, and immunoglobulin A were measured using a KHB-1280 fully automatic biochemistry analyzer (Shanghai Kehua Bioengineering Co., Ltd., Shanghai, China). Superoxide dismutase, malondialdehyde, total antioxidant capacity, and glutathione peroxidase were measured using a L-3180 semi-automatic biochemistry analyzer (Shanghai Kehua Bioengineering Co., Ltd., Shanghai, China). Progesterone, estradiol, prolactin, cortisol, non-esterified fatty acids, β-hydroxybutyric acid, interleukin-1β, interleukin-6, interleukin-10, and tumor necrosis factor were measured in serum by enzyme-linked immunosorbent assay (ELISA) kits, using an ST-360 enzyme labeler (Shanghai Kehua Bioengineering Co., Ltd., Shanghai, China), according to the manufacturer’s instructions. Simply, the antibodies were added to the sample and bound to the enzyme complex, and the contents were detected via color development. ELISA kits were purchased from Beijing Jinhai Kecum Biotechnology Development Co. (Beijing, China).

Milk samples were collected from 60 cows, with 15 randomly selected from each group. Colostrum, transitional milk, and regular milk samples were taken on days 1, 4, 7, and 14 postpartum. Fifty milliliters of milk were collected each time, mixed with potassium dichromate, and stored at 4 °C. The samples were used to complete the Dairy Herd Improvement (DHI) analysis. The daily milk production of the test cows was recorded by the automatic milk analysis system of the farm, and the milk samples were tested for lactose, fat, milk protein, urea nitrogen content, and somatic cell count at the Milk Quality Inspection Station of Beijing Dairy Cattle Center by using the Milk Ingredient Somatic Cell Count In-Line Tester (YQ1-57).

### 2.3. Statistical Analysis

All data were expressed as mean ± standard error of the mean (SEM). The data were analyzed using one-way ANOVA, followed by Duncan’s multiple tests to assess the significance of differences between groups. To compare between incidence and mating pregnancy rate, the Chi-squared test was performed. The statistical analyses were performed using GraphPad Prism 9.0.0 software. *p* < 0.05 was considered a statistically significant difference.

## 3. Results

### 3.1. Effect of AKG Supplementation on Metabolism and Liver Function of Cows in Their Periparturient

**Figure 1:**
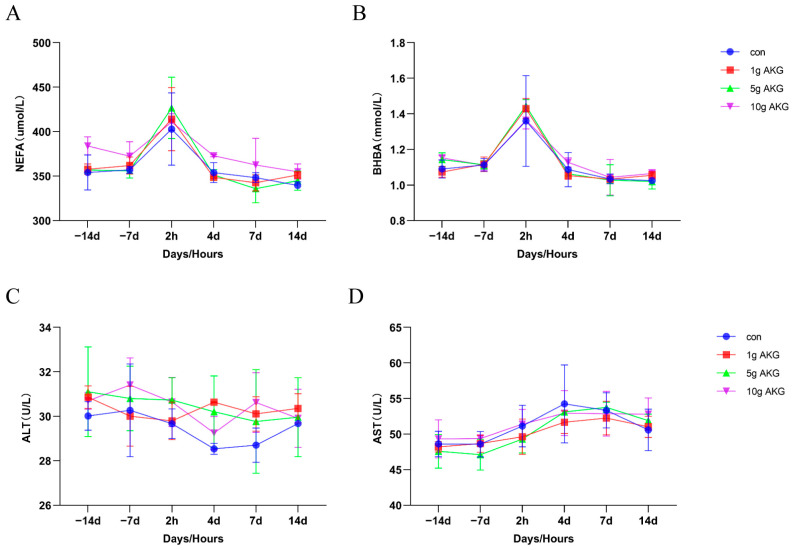
Effect of AKG supplementation on metabolism and liver function of cows in their periparturient period. (**A**) Serum non-esterified fatty acid (NEFA) levels, (**B**) serum β-hydroxybutyric acid (BHBA) levels, (**C**) serum alanine aminotransferase (ALT) levels, and (**D**) serum aspartate aminotransferase (AST) levels. Days −14, −7: peri-delivery days; 2 h, 4 d, 7 d, and 14 d: post-delivery hours and days. Data are expressed as mean ± SEM (*n* = 5).

The results show that AKG supplementation had no significant effects on serum non-esterified fatty acids (NEFA), beta-hydroxybutyric acid (BHBA), alanine aminotransferase (ALT), and aspartate aminotransferase (AST) compared to the control group (*p* > 0.05). (Figure 1).

### 3.2. Effect of AKG Supplementation on Blood Biochemical Indexes of Cows in Their Periparturient Period

**Figure 2:**
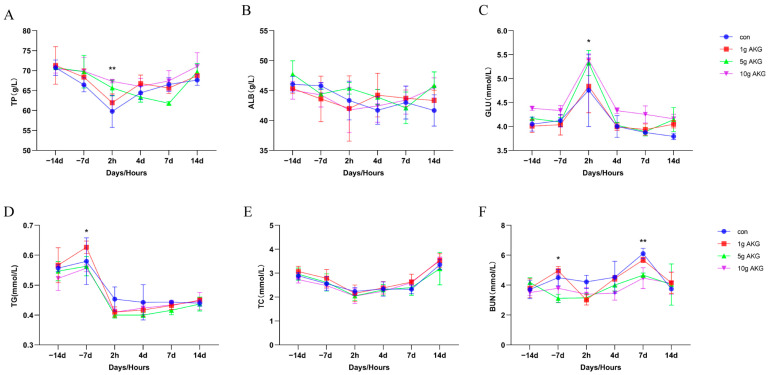
Effect of AKG supplementation on blood biochemical indexes of cows in their periparturient period. (**A**) Serum total protein (TP) levels, (**B**) serum albumin (ALB) levels, (**C**) serum glucose (GLU) levels, (**D**) serum triglyceride (TG) levels, (**E**) serum total cholesterol (TC) levels, and (**F**) serum urea nitrogen (BUN) levels. Days −14, −7: peri-delivery days; 2 h, 4 d, 7 d, and 14 d: post-delivery hours and days. Data are expressed as mean ± SEM (*n* = 5). * *p* < 0.05, ** *p* < 0.01 between groups.

The results show that at 2 h after delivery, serum total protein (TP) levels were significantly higher in the 5 g and 10 g AKG groups than in the control group (5 g AKG: 65.67 ± 1.97, 10 g AKG: 67.30 ± 0.38 vs. control: 59.77 ± 4.02 g/L) (*p* < 0.05) (Figure 2A). The serum albumin (ALB) levels of the groups had no significant differences (*p* > 0.05) (Figure 2B). However, the serum glucose (GLU) levels were significantly increased in all of the groups at 2 h after delivery compared to other time points, in which the GLU levels in the 5 g and 10 g AKG groups were even higher than that in the control groups (5 g AKG: 5.33 ± 0.26, 10 g AKG: 5.39 ± 0.10 vs. control:4.75 ± 0.75 mmol/L) (*p* < 0.05) (Figure 2C). The changes in the levels of serum triglyceride (TG) in the cows of the four groups tended to be consistent, reaching a maximum at 7 days before parturition and then decreasing. After 7 days of feeding, serum TG levels were significantly lower in the 5 g AKG and 10 g AKG groups than in the 1 g AKG group (5 g AKG: 0.56 ± 0.03, 10 g AKG: 0.55 ± 0.01 vs. 1 g AKG: 0.63 ± 0.02 mmol/L) (*p* < 0.05) (Figure 2D). The serum total cholesterol (TC) levels showed no significant differences between all of the time points and groups (*p* > 0.05) (Figure 2E). The results also show that after 7 days of feeding, serum urea nitrogen (BUN) levels were significantly higher in the 1 g AKG group than in the 5 g AKG group (1 g AKG: 4.96 ± 0.28 vs. 5 g AKG: 3.15 ± 0.28 mmol/L) (*p* < 0.05), and after 7 days of parturition in the dairy cows, serum BUN levels were significantly lower in the 5 g AKG group than in the control group in both the 5 g AKG group and the 10 g AKG group (control. 6.11 ± 0.35 vs. 5 g AKG: 4.67 ± 0.14,10 g AKG: 4.47 ± 0.69 mmol/L) (*p* < 0.05) (Figure 2F).

### 3.3. Effect of AKG Supplementation on Serum Immunological Indexes of Cows in Their Periparturient Period

**Figure 3:**
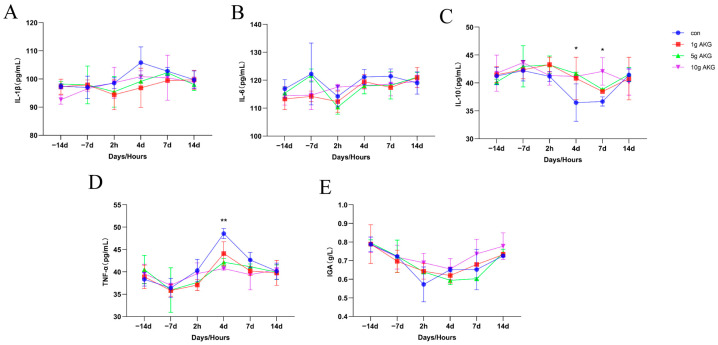
Effect of AKG supplementation on serum immunological indexes of cows in their periparturient period. (**A**) Serum interleukin 1β (IL-1β) levels, (**B**) serum interleukin 6 (IL-6) levels, (**C**) serum interleukin 10 (IL-10) levels, (**D**) serum tumor necrosis factor α (TNF-α) levels, (**E**) serum immunoglobulin A (IgA) levels. Days −14, −7: peri-delivery days; 2 h, 4 d, 7 d, and 14 d: post-delivery hours and days. Data are expressed as mean ± SEM (*n* = 5), * *p* < 0.05, ** *p* < 0.01 between groups.

The results show that the serum interleukin 1β (IL-1β) (Figure 3A), interleukin 6 (IL-6) (Figure 3B), and immunoglobulin A (IgA) (Figure 3E) levels exhibited no significant differences between the time points and groups (*p* > 0.05). However, on day 4 post-delivery, the levels of interleukin 10 (IL-10) in 5 g and the 10 g AKG groups were significantly higher than in the control group (5 g AKG: 41.67 ± 0.25, 10 g AKG: 41.18 ± 0.11 vs. control: 36.48 ± 3.35 pg/mL). On days 7 post-delivery, the levels of IL-10 in 10 g AKG groups were significantly higher than in the control group (10 g AKG: 42.07 ± 2.46 vs. control: 36.68 ± 0.82 pg/mL) (*p* < 0.05) (Figure 3C) (*p* < 0.05) (Figure 3C). On day 4 post-delivery, the serum tumor necrosis factor alpha (TNF-α) was significantly increased compared to other time points and this increase was significantly lower in the 5 g and 10 g AKG groups than in the control group (5 g AKG: 42.16 ± 0.84,10 g AKG: 40.75 ± 0.32 vs. control: 48.58 ± 1.12 pg/mL) (*p* < 0.01) (Figure 3D).

### 3.4. Effect of AKG Supplementation on Serum Antioxidant Capacity of Cows in Their Periparturient Period

**Figure 4:**
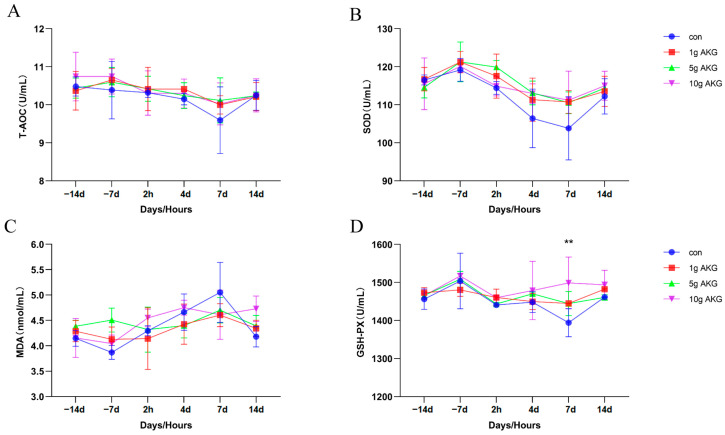
Effect of AKG supplementation on serum antioxidant capacity of cows in their periparturient period. (**A**) Serum total antioxidant capacity (T-AOC) levels, (**B**) serum superoxide dismutase (SOD) levels, (**C**) serum malondialdehyde (MDA) levels, (**D**) serum glutathione peroxidase (GSH-PX) levels. Days −14, −7: peri-delivery days; 2 h, 4 d, 7 d, and 14 d: post-delivery hours and days. Data are expressed as mean ± SEM (*n* = 5), ** *p* < 0.01 between groups.

The serum total antioxidant capacity (T-AOC) (Figure 4A) and superoxide dismutase (SOD) (Figure 4B) levels were slightly higher in the AKG groups than in the control group, but alterations did not reach statistically significant differences (*p* > 0.05). The malondialdehyde (MDA) levels gradually increased after delivery and reached their peak on day 7, and even the MDA levels in the 5 g and 10 g AKG groups were lower than in the control group, but no statistically significant differences were detected between the groups (*p* > 0.05) (Figure 4C). The levels of glutathione peroxidase (GSH-PX) were overall higher in all the AKG groups than in the control group after delivery, but only the 10 g AKG group on day 7 after delivery showed a significant difference compared to the control group (10 g AKG: 1498.90 ± 67.91 vs. control: 1394.22 ± 36.99 U/mL) (*p* < 0.05) (Figure 4D).

### 3.5. Effect of AKG Supplementation on Serum Reproductive Hormones of Cows in Their Periparturient Period

**Figure 5:**
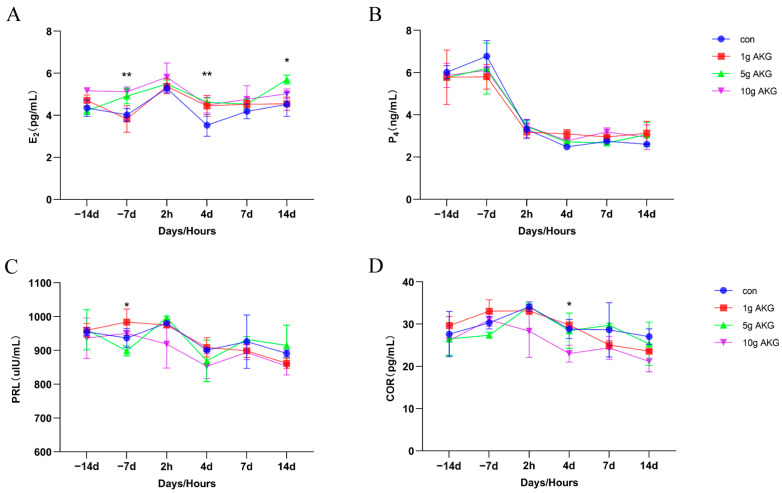
Effect of AKG supplementation on serum reproductive hormones of cows in their periparturient period. (**A**) Serum estradiol (E_2_) levels, (**B**) serum progesterone (P_4_) levels, (**C**) serum prolactin (PRL) levels, and (**D**) serum cortisol (COR) levels. Days −14, −7: peri-delivery days; 2 h, 4 d, 7 d, and 14 d: post-delivery hours and days. Data are expressed as mean ± SEM (*n* = 5), * *p* < 0.05, ** *p* < 0.01 between groups.

Serum estradiol (E_2_) levels varied considerably in periparturient cows, with overall E2 levels in the control group being lower than those in the experimental group. After 7 days of feeding, E_2_ levels were significantly higher in the 5 g AKG and 10 g AKG groups than in the control and 1 g AKG groups (5 g AKG: 4.92 ± 0.37, 10 g AKG: 5.12 ± 0.23 vs. control: 4.02 ± 0.30, 1 g AKG: 3.82 ± 0.63 pg/mL) (*p* < 0.05). At 4 and 14 days after parturition, E2 levels were significantly higher in the 5 g AKG group than in the control group (4 d, 5 g AKG: 4.63 ± 0.20 vs. control: 3.53 ± 0.52 pg/mL) (14 d, 5 g AKG: 5.70 ± 0.21 vs. control: 4.52 ± 0.57 pg/mL) (*p* < 0.05) (Figure 5A). The serum progesterone (P_4_) levels were significantly reduced right after delivery in all groups, and no significant differences were observed between the groups at all time points (*p* > 0.05) (Figure 5B). The prolactin (PRL) levels were not significantly different between the groups at most time points; only at 7 days into the periparturient period, the prolactin level in the 1 g AKG group was significantly higher than that in the 5 g AKG group (1 g AKG: 29.42 ± 1.49 vs. 10 g AKG: 21.09 ± 1.36 pg/mL) (*p* < 0.05) (Figure 5C). The serum cortisol (COR) level in the 10 g AKG group appeared lower after delivery; but only on the 4th day post-delivery did this level show a significant difference compared to the control and other groups (*p* < 0.05) (Figure 5D).

### 3.6. Effect of AKG Supplementation During Periparturient Period on Incidence of Postpartum Diseases and Postpartum Mating Pregnancy Rates

The incidence of postpartum diseases in each group is listed in Table 1. The incidence of postpartum diseases in the AKG groups was lower than that in the control group, and the lowest incidence was observed in the 5 g and 10 g AKG groups, which was 26.7%, vs. 53.5% in the control group (*p* < 0.05). Then, the effect of a pre-partum dietary addition of AKG on the postpartum pregnancy rate was observed. The addition of AKG positively affected the pregnancy rate of dairy cows in the first postpartum mating period and the cumulative pregnancy rate of the two mating periods. In particular, cows fed the 5 g and 10 g AKG groups had significantly higher (*p* < 0.05) first mating pregnancy rates and cumulative pregnancy rates than did the control group (Table 2).

### 3.7. Effect of AKG Supplementation During Periparturient Period on Calf Birth Weight and Open Days

**Figure 6:**
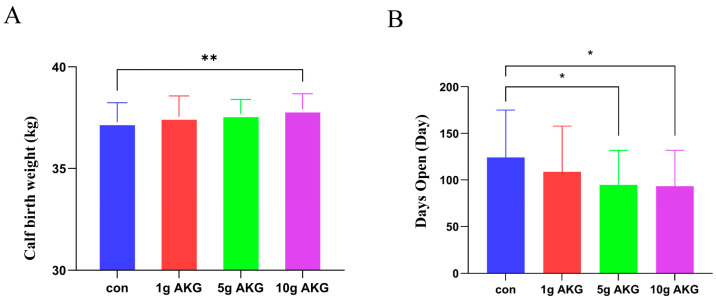
Effect of AKG supplementation during periparturient period on calf birth weight and interval between postpartum days to successful breading. (**A**) Newborn calf weight, (**B**) interval between postpartum to next successful breading. Data were expressed as mean ± SEM (*n* = 45), * *p* < 0.05, ** *p* < 0.01 vs. control group.

The results show that the calf birth weight in the 10 g AKG group was significantly higher than those in the control and other groups (10 g AKG: 37.90 ± 0.77 kg vs. control: 37.28 ± 0.96 kg) (*p* < 0.05) (Figure 6A). In addition, the results also show that the open days of the cows were significantly shorter in the 5 and 10 g AKG groups than in the control group (*p* < 0.05) (Figure 6B).

### 3.8. Effect of AKG Supplementation During Periparturient Period on Lactation Performance

**Figure 7:**
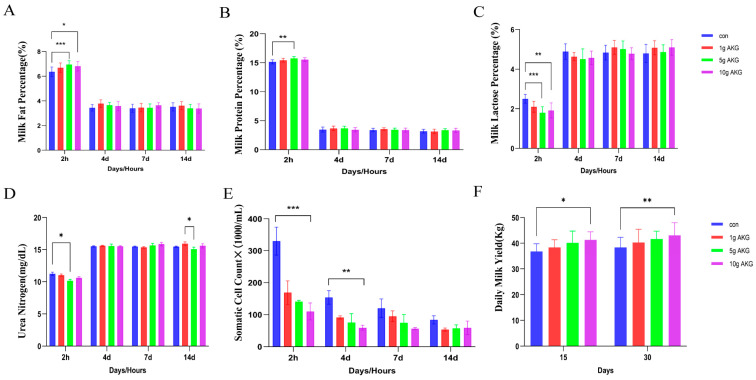
Effect of AKG supplementation during periparturient period on milk composition and daily milk yield. (**A**) Milk fat percentages, (**B**) milk protein percentages, (**C**) milk lactose percentages, (**D**) milk urea nitrogen levels, (**E**) milk somatic cell counts, and (**F**) daily milk yields. The measurements were taken at 2 h, 4 d, 7 d, and 14 d postpartum. Data are expressed as mean ± SEM (*n* = 5), * *p* < 0.05, ** *p* < 0.01, *** *p* < 0.001 vs. control group.

The results showed that at 2 h postpartum, the milk fat percentage of the colostrum was significantly higher in both the 5 and 10 g AKG groups than in the control group (5 g AKG: 6.95 ± 0.31,10 g AKG: 6.81 ± 0.38, control: 6.36 ± 0.40%) (*p* < 0.05) (Figure 7A). Thereafter, at 4, 7, and 14 days postpartum, the milk fat percentages were stabilized at relatively low levels compared to the 2 h time point, with no significant differences between the groups (Figure 7A). The milk protein percentage exhibited a similar pattern as milk fat. At 2 h postpartum, the colostrum lactose percentage was significantly lower in both the 5 and 10 g AKG groups than in the control group (5 g AKG: 15.68 ± 0.26 vs. control: 15.16 ± 0.35%) (*p* < 0.05) (Figure 7B). At 2 h postpartum, the milk lactose percentage of the colostrum was significantly higher in both the 5 and 10 g AKG groups than in the control group (5 g AKG: 1.98 ± 0.16,10 g AKG: 0.16 ± 0.22 control: 2.5 ± 0.22%) (*p* < 0.05). Thereafter, on 4, 7, and 14 days postpartum, milk fat percentages were stabilized at relatively high levels compared to the 2 h time point with significant differences (Figure 7C). At 2 h postpartum, the urea nitrogen of the colostrum was significantly lower in the 5 g AKG group than in the control group (5 g AKG: 11.23 ± 0.74 vs. control: 10.15 ± 0.68 mg/dL) (*p* < 0.05) (Figure 7D), while on the days 4, 7, and 14 postpartum, the urea nitrogen levels were stabilized at relatively high levels compared to 2 h postpartum, with no significant differences between the groups, except that on day 14, the urea nitrogen in standing milk was significantly lower in the 5 g AKG group than in the 1 g AKG group (5 g AKG: 15.07 ± 1.08 vs. 1 g AKG: 15.93 ± 0.99 mg/dL) (*p* < 0.05). At 2 h postpartum, the somatic cell count in the colostrum was significantly lower in all AKG groups compared to the control group (1 g AKG: 169.00 ± 36.77, 5 g AKG: 141.00 ± 4.24, 10 g AKG: 110.33 ± 26.58 × 1000/mL vs. control: 330.00 ± 43.84 × 1000/mL) (*p* < 0.0001). After that, the somatic cell count was significantly reduced in all groups. However, all AKG groups continued to have a lower somatic cell count than the control group. Notably, at day 4 postpartum, the somatic cell count in the standing milk of the 10 g AKG group remained significantly lower than that of the control group (10 g AKG: 59.50 ± 7.78 × 1000/mL vs. control: 154.00 ± 21.21 × 1000/mL) (*p* < 0.05). At other time points, no significant differences were observed between the groups (Figure 7E). The daily milk production on days 15 and 30 post-partum was measured. The results showed that milk production was higher in all AKG groups than in the control groups, but only the 10 g AKG group reached a significant difference compared to the control group (15 days: 41.30 ± 3.16 vs. 36.80 ± 3.03 kg; 30 days: 43.10 ± 4.93 vs. 38.40 ± 3.91 kg) (*p* < 0.05) (Figure 7F).

## 4. Discussion

Evidence shows that NEB is strongly associated with immunosuppression in cows, which is the major factor for the high prevalence of postpartum diseases, especially mastitis [20,21]. Usually, the elevated plasma concentrations of NEFA and BHBA, which are the metabolites of fat, are indicators of the potential NEB status in dairy cows [22,23]. Our results showed that at 2 h postpartum, the serum NEFA and BHBA were significantly elevated in the cows, and this confirmed their NEB occurrence. However, AKG supplementation in this study failed to improve the levels of serum NEFA and BHBA in the cows, indicating that the direct effect of AKG on improving the NEB was limited. In addition, AKG supplementation did not significantly modify the serum cholesterol and triglyceride levels, and these results further suggest that AKG probably would not be involved in fat metabolism in the periparturient period of cows. Our results showed that AKG supplementation indeed somehow impacted the protein and glucose metabolism, i.e., to lower the serum protein levels and urea nitrogen levels of milk and increase the serum glucose level compared to the control group during the postpartum period. Similar results have been reported in sheep [17]. On the other hand, AKG supplementation significantly increased the calves’ newborn weights, suggesting that AKG promotes fetal growth and development by optimizing maternal nutritional status. This seems contradictory to the observation that AKG supplementation has a limited beneficial effect on lipid metabolism and NEB. It also suggests that other beneficial mechanisms might be involved in AKG supplementation for mother cows.

We noticed that reproductive hormones, including E_2_, P_4_, PRL, and COR, play key roles in reproductive health and lactation regulation in periparturient dairy cows [24]. In this study, it was found that AKG supplementation had a profound effect on reproductive hormone levels during the periparturient period. The AKG-supplemented group exhibited an overall higher level of E_2_ than did the control group, and the higher level of E_2_ after parturition favors the promotion of estrus in cows [25]. PRL plays an important role in mammary gland development and lactation initiation [26]. The PRL in the 10 g AKG group was lower than that in the control group even without a significant difference. This low level of PRL had a promoting effect on the synthesis of estrogen and progesterone in the ovaries, which was more favorable for estrus in postpartum cows. COR is essential for neonatal survival as well as lactation and labor [27]. However, AKG supplementation has no significant effects on it. The results suggest that AKG may regulate hormone secretion to improve energy metabolism; therefore, this treatment balances the NEB during the periparturient period and improves the reproductive function of cows [28,29]. It was observed in the current study that AKG supplementation significantly increased the mating pregnancy rate and shortened the postpartum mating interval.

Oxidative stress is another risk factor for reproductive physiology; especially during the periparturient period, the immune function and inflammatory responses of dairy cows are vulnerable to oxidative stress [30,31]. In the present study, it was the case that AKG supplementation had the capacity to increase the anti-inflammatory and antioxidant activities of the cows. Specifically, this treatment significantly increased the serum levels of IL-10 while lowering the pro-inflammatory factors TNF-α and interleukin 1β compared to the control group. IL-10, as a key anti-inflammatory cytokine, can effectively inhibit the expression of pro-inflammatory genes, thereby reducing the inflammatory response [32]. This result is consistent with a previous report that AKG enhanced immune response [33]. The GSH-PX is a major antioxidant enzyme that detoxifies cell-generated hydrogen peroxide. AKG supplementation significantly elevated the level of GSH-PX. This further suggests that AKG is able to alleviate the oxidative stress of cows during the periparturient period by enhancing the antioxidant defense system, which is in line with the promotion of antioxidant capacity by AKG in the literature [34,35]. Therefore, AKG supplementation reduced the incidence of periparturient diseases, including mastitis, as observed in this study. This should be attributed to the fact that the antioxidative activity of AKG suppressed the inflammatory response and improved the immune function of the cows [36,37].

Milk production in dairy cows is affected by a variety of factors, including breed, stage of lactation, litter size, light condition, disease, feed composition, and nutritional status [38,39]. In the present study, milk fat and protein percentages of colostrum in the cows in the 5 and 10 g AKG groups were significantly higher than those of the control group, and the number of colostrum somatic cells was significantly reduced compared to the control group. This suggests that AKG not only improves the quality of the colostrum, but also the mammary health of dairy cows. The reduction in somatic cell counts is usually associated with a reduction in mammary gland inflammation. In addition, we observed in this study that AKG improved postpartum lactation in cows. All of this may be attributed to AKG being an important energy metabolic intermediator, improving energy metabolism, enhancing antioxidant capacity, and regulating the immune function of cows, which is consistent with the results reported in pigs [40,41,42].

## 5. Conclusions

The present study showed that supplementation with different doses of AKG during the peripartum period had a significant effect on the health and reproductive factors of cows. It improved the lactation performance of the cows, including an increase in the milk fat, protein percentage, and daily milk yield, as well as a reduction of the milk somatic cell count. Most importantly, AKG supplementation not only increased calf birth weight, but also shortened the interval between the postpartum period to the next successful *breeding* period. These beneficial effects of AKG supplementation during the peripartum period may be attributed to AKG improving the anti-inflammatory and antioxidant capacity, reproductive hormone secretion, and relief of the NEB of the cows.

## Figures and Tables

**Table 1:** Effect of AKG supplementation during periparturient period on incidence of postpartum diseases.

Items	Control	1 g	5 g	10 g
Total	45	45	45	45
Retained placenta	7	3	3	0
Metritis	3	1	0	0
Mastitis	12	10	8	8
Postpartum paralysis	0	0	0	0
Ketosis	1	0	0	1
Diarrhea	1	3	1	3
Healthy	21	28	33	33
Affected	24	17	12	12
Incidence rate (%)	53.3 b	37.8 ab	26.7 a	26.7 a

Note: different lowercase letters indicate significant differences (*p* < 0.05), and same letter indicates non-significant differences (*p* > 0.05) between groups.

**Table 2:** Effect of AKG supplementation during periparturient period on postpartum mating pregnancy rates.

Items	Control	1 g	5 g	10 g
Total	45	45	45	45
First insemination pregnancy number	10	16	19	23
First insemination pregnancy rate (%)	22.22 a	35.56 b	42.22 b	51.51 b
Second insemination pregnancy number	15	25	29	30
Second insemination pregnancy rate (%)	33.33 a	55.56 b	61.70 b	66.67 b

Note: different lowercase letters indicate significant differences (*p* < 0.05), and same letter indicates non-significant differences (*p* > 0.05) between groups.

## Data Availability

The data presented in this study are available upon request from the corresponding author.
